# Differential Mortality Risk by Age in Males and Females With Steatotic Liver Disease

**DOI:** 10.1002/mco2.70677

**Published:** 2026-04-05

**Authors:** Taotao Yan, Nicholas Chien, Vy H. Nguyen, Isaac Le, Surya Teja Gudapati, Angela Chau, Xinrong Zhang, Scott Barnett, Sovann Linden, Linda Henry, Ramsey Cheung, Mindie H. Nguyen

**Affiliations:** ^1^ Division of Gastroenterology and Hepatology Stanford University Medical Center Palo Alto California USA; ^2^ Department of Infectious Diseases The First Affiliated Hospital of Xi'an Jiaotong University Xi'an Shaanxi China; ^3^ Division of Gastroenterology and Hepatology Veterans Affairs Palo Alto Healthcare System Palo Alto California USA; ^4^ Department of Epidemiology and Population Health Stanford University Medical Center Palo Alto California USA; ^5^ Stanford Cancer Institute Stanford University Medical Center Palo Alto California USA

**Keywords:** age, liver‐related mortality, MASLD, nonliver‐related mortality, overall mortality, sex differences

## Abstract

Metabolic dysfunction‐associated steatotic liver disease (MASLD) is a major cause of premature mortality, but data on sex differences in mortality remain limited. We compared the overall, nonliver‐related, and liver‐related mortality rates per 1000 person‐years in MASLD patients by sex. Propensity score matching (PSM) yielded 3579 pairs of females and males with balanced characteristics from a cohort of 8517 MASLD patients (53.1% female, 46.6% male) seen at Stanford University Medical Center (1995–2023). In the total PSM cohort, the overall (12.68 vs. 12.92), nonliver‐related (11.43 vs. 11.60), and liver‐related (1.25 vs. 1.32) mortality rates were similar between males and females. However, in age‐stratified analyses, females had higher overall (7.99 vs. 4.95, *p *= 0.02) and nonliver‐related (7.20 vs. 4.71, *p *= 0.05) mortality rates among younger (≤50 years) patients, with opposite direction among the older group with higher overall (21.40 vs. 16.51, *p *= 0.02) and nonliver‐related (19.02 vs. 14.80, *p *= 0.04) mortality rates in males. In Cox regression analyses, male sex was associated with lower risks of overall and nonliver‐related mortality (adjusted hazard ratio [aHR] 0.59 and 0.61) among patients ≤50 years, but with higher risks among those >50 years (aHR 1.32 and 1.30). Sex and age should be considered in the management strategies for people with MASLD.

## Graphical abstract

Data on the impact of sex for overall and cause‐specific mortality in patients with metabolic dysfunction‐associated steatotic liver disease (MASLD) is limited. Among older (>50 years) people with MASLD, males had about 40% higher risk of overall and nonliver related mortality, while the opposite was observed with females having about 30% higher risk among younger (≤50 years) people with MASLD. Significant sex differences exist in mortality risks of people with MASLD and should be taken into account in the management of MASLD.

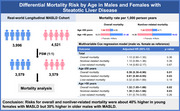

## 1 Introduction

Metabolic dysfunction‐associated steatotic liver disease (MASLD) is a major global health problem affecting over 30% of the population worldwide [1], associating with increased liver‐related and nonliver‐related mortality [2, 3]. Prior studies have reported associations of male sex and age with risk of MASLD [1], but data on the impact of sex on the natural history of MALSD are currently limited.

Liver disease may exhibit sex‐based characteristics due to the presence of estrogen and androgen receptors on hepatocytes, as well as its different response to sex hormones [4]. Sex disparities have been reported for various liver diseases such as hepatocellular carcinoma (HCC) and chronic hepatitis B. For HCC, males generally have higher risks for HCC development than females [5]. For chronic hepatitis B, females have better virological and clinical responses to nucleos(t)ide analogue therapy than males [6].

For MASLD, two prior studies from South Korea compared mortality between people with MASLD and those without MASLD [3, 7]. The studies explored sex differences in overall and cardiovascular (CVD)‐related mortality between the MASLD versus non‐MASLD groups in a subgroup analysis by sex and found higher mortality in only females. However, the studies did not focus on sex differences among people with MASLD in general, and the analysis did not account for potential confounders in the subgroup analyses by sex. On the other hand, a recent study that investigated sex disparities in MASLD using the National Health and Nutrition Examination Survey data reported opposite findings where females were found to have lower all‐cause and CVD‐related mortality [8]. This differences in findings may be due to differences in the different study inclusion criteria in different studies, lack of adequate adjustment for potential confunders, or limited data on liver‐related mortality.

To fill in this data gap, we investigated the impact of sex on overall, nonliver‐related, and liver‐related mortality in a large real‐world cohort of patients with MASLD with long‐term follow‐up data using propensity score matching (PSM) method to balance patient characteristics between the male and female groups.

## 2 Results

### 2.1 Total (Unmatched) Study Population Characteristics and Mortality Analyses

**FIGURE 1:**
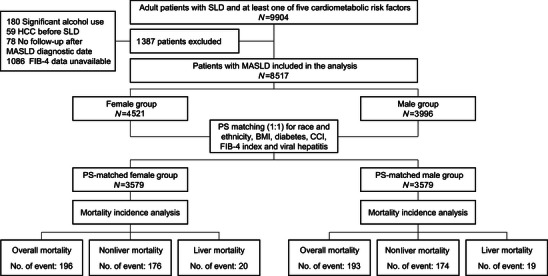
Study flowchart. BMI, body mass index; CCI, Charlson Comorbidity Index; FIB‐4, fibrosis‐4; HCC, hepatocellular carcinoma; MASLD, metabolic dysfunction‐associated steatotic liver disease; PS, propensity‐score; SLD, steatotic liver disease.

Baseline characteristics of the study population are presented in **Table** **1**. Overall, 8517 adult patients (4521 females and 3996 males) with MASLD met the study inclusion criteria and were included in the analysis (Figure 1). Before PSM, compared with females, males were younger (49.9 vs. 51.8 years), more Asian (27.30 vs. 19.86%) but less Hispanic (17.82 vs. 30.15%), less likely to be obese (55.91 vs. 63.33%) or diabetic (29.48 vs. 35.85%) but more likely to have hyperlipidemia (53.45 vs. 46.43%), all *p *< 0.001. Males compared with females were also more likely to have chronic kidney disease (CKD) (10.06 vs. 6.66%, *p *< 0.001) but less likely to have nonliver cancer (18.99 vs. 21.46%, *p *= 0.01). Compared with females, males had a higher proportion of viral hepatitis (6.23 vs. 4.07%, *p *< 0.001), but comparable for cirrhosis (6.41 vs. 5.60%, *p *= 0.13). However, the mean Charlson Comorbidity Index (CCI) was higher in females compared with males (2.87 vs. 2.74, *p *= 0.01).

**TABLE 1:** Characteristics of patients with MASLD by sex.

Characteristic	Before PS matching patients, no. (%)				After PS matching^a^ patients, no. (%)			
	Female (*N* = 4521)	Male (*N* = 3996)	*p* value	SMD	Female (*N* = 3579)	Male (*N* = 3579)	*p* value	SMD
Age, years	51.8 ± 15.9	49.9 ± 15.2	<0.001	0.12	52.9±15.7	50.1±15.3	<0.001	0.18
Race and ethnicity			<0.001	0.31			0.62	0.04
White	1787 (39.53)	1800 (45.05)			1651 (46.13)	1645 (45.96)		
Black	125 (2.76)	81 (2.03)			74 (2.07)	80 (2.24)		
Asian	898 (19.86)	1091 (27.30)			851 (23.78)	884 (24.70)		
Hispanic	1363 (30.15)	712 (17.82)			712 (19.89)	710 (19.84)		
Other	348 (7.70)	312 (7.81)			291 (8.13)	260 (7.26)		
BMI			<0.001	0.18			0.47	0.03
Normal	346 (7.65)	264 (6.61)			276 (7.71)	258 (7.21)		
Overweight	1312 (29.02)	1498 (37.49)			1151 (32.16)	1193 (33.33)		
Obese	2863 (63.33)	2234 (55.91)			2152 (60.13)	2128 (59.46)		
Diabetes	1621 (35.85)	1178 (29.48)	<0.001	0.14	1169 (32.66)	1168 (32.63)	1.00	0.001
Hypertension	2154 (47.64)	1899 (47.52)	0.93	0.002	1721 (48.09)	1758 (49.12)	0.40	0.02
Hyperlipidemia	2099 (46.43)	2136 (53.45)	<0.001	0.14	1680 (46.94)	1960 (54.76)	<0.001	0.16
CVD	450 (9.95)	409 (10.24)	0.69	0.01	345 (9.64)	388 (10.84)	0.10	0.04
CKD	301 (6.66)	402 (10.06)	<0.001	0.12	244 (6.82)	371 (10.37)	<0.001	0.13
Nonliver cancer	970 (21.46)	759 (18.99)	0.01	0.06	807 (22.55)	699 (19.53)	0.002	0.07
CCI	2.87 ± 2.26	2.74 ± 2.29	0.01	0.06	2.84 ± 2.23	2.81 ± 2.36	0.50	0.02
Viral hepatitis	184 (4.07)	249 (6.23)	<0.001	0.10	169 (4.72)	185 (5.17)	0.41	0.02
Cirrhosis	253 (5.60)	256 (6.41)	0.13	0.03	212 (5.92)	214 (5.98)	0.96	0.002
ALT^b^, U/L	38.0 (25.0, 65.0)	52.0 (34.0, 82.0)	<0.001	0.43	38.0 (25.5, 65.0)	53.0 (35.0, 84.0)	0.001	0.46
AST^b^, U/L	28.0 (20.0, 46.0)	32.0 (23.0, 48.0)	<0.001	0.21	29.0 (21.0, 47.0)	32.0 (24.0, 48.0)	<0.001	0.16
FIB‐4 index			0.04	0.06			1.00	0.002
<1.3	3093 (68.41)	2633 (65.89)			2338 (65.33)	2338 (65.33)		
1.3–2.67	1019 (22.54)	958 (23.97)			894 (24.98)	896 (25.03)		
>2.67	409 (9.05)	405 (10.14)			347 (9.70)	345 (9.64)		
Albumin, g/dL	3.94±0.56	4.12 ± 0.57	<0.001	0.31	3.96 ± 0.56	4.11 ± 0.58	<0.001	0.26
Bilirubin^b^, mg/dL	0.5 (0.3, 0.6)	0.6 (0.4, 0.8)	<0.001	0.18	0.5 (0.3, 0.6)	0.6 (0.4, 0.8)	<0.001	0.17
Creatinine^b^, mg/dL	0.72 (0.62, 0.86)	0.98 (0.85, 1.10)	<0.001	0.56	0.73 (0.63, 0.88)	0.98 (0.84, 1.10)	<0.001	0.54

Abbreviations: ALT, alanine aminotransferase; AST, aspartate aminotransferase; BMI, body mass index; CCI, Charlson Comorbidity Index; CKD, chronic kidney disease; CVD, cardiovascular disease; FIB‐4, fibrosis‐4; MASLD, metabolic dysfunction‐associated steatotic liver disease; PS, propensity‐score; SMD, standardized mean difference.

^a^ Matched for race and ethnicity, BMI, diabetes, Charlson Comorbidity Index, FIB‐4 Index, and viral hepatitis.

^b^ Median (IQR).

In this total unmatched cohort, 256 females and 206 males died after a total follow‐up of 19,016 person‐years (PY) for females and 17,209 PY for males. Specifically, 233 nonliver‐related deaths were confirmed in females and 183 in males, while 23 liver‐related deaths occurred in females and 23 in males. Males as compared with females had comparable overall (11.97 vs. 13.46), nonliver‐related (10.63 vs. 12.25), and liver‐related (1.34 vs. 1.21) mortality rates per 1000 PY, all *p *> 0.05 (Table S1). The 5‐year cumulative incidence in males and females were 6.2 versus 6.0% for overall mortality, 5.5 versus 5.6% for nonliver‐related mortality, and 0.70 versus 0.41% for liver‐related mortality, respectively (Figure S1).

### 2.2 Matched Study Population Characteristics and Mortality Analyses

After PSM, 3579 pairs of male and female patients with balanced baseline characteristics were included in the matched MASLD cohort (Figure 1 and Table 1). The distribution of propensity scores after PSM between female and male groups was highly overlapping, indicating a good matching quality (Figure S2). While males and females showed statistically significant differences in age, hyperlipidemia, CKD, and laboratory tests, the mean CCI (2.81 vs. 2.84) and fibrosis‐4 (FIB‐4) index were nearly identical between males and females, with *p* values of 0.5 for CCI and 1.0 for FIB‐4 index, respectively. Aspartate aminotransferase (AST), albumin, and bilirubin were similar between males and females. Since males generally have higher alanine aminotransferase (ALT) and creatinine compared with females under physiological conditions [9, 10], we considered the slightly higher ALT and creatinine in males compared with females in our study to be not of major clinical significance.

**FIGURE 2:**
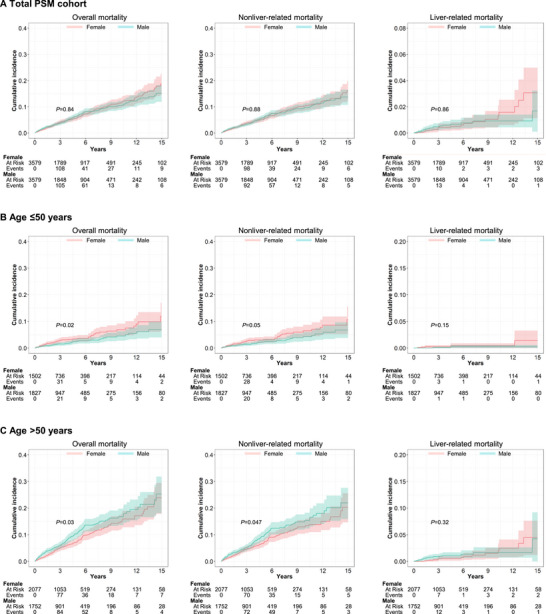
Cumulative incidence of mortality in patients with MASLD by sex in the (A) total PSM cohort, (B) subgroup of patients aged ≤50 years and (C) subgroup of patients aged >50 years within the PSM cohort. MASLD, metabolic dysfunction‐associated steatotic liver disease; PSM, propensity score matching.

In this matched cohort, death was confirmed in 196 females and 193 males over a total follow‐up of 15,170 PY for females and 15,225 PY for males. Of these, mortality in 176 females and 174 males were identified as nonliver‐related mortality, while mortality in 20 females and 19 males were identified as liver‐related mortality. The overall (12.68 vs. 12.92 per 1000 PY), nonliver‐related (11.43 vs. 11.60 per 1000 PY), and liver‐related (1.25 vs. 1.32 per 1000 PY) mortality rates were similar between males and females, respectively, all *p *> 0.05 (Table 2). The 5‐year cumulative incidence between males and females were also similar at 6.5 versus 5.7% for overall mortality, 5.9 versus 5.2% for nonliver‐related mortality, and 0.67 versus 0.51% for liver‐related mortality, respectively (Figure 2A).

**TABLE 2:** Mortality rates in patients with MASLD in the PSM cohort.

Outcome	Group	Patient no.	Person‐years	Event, *n*	Mortality rate per 1000 person‐years (95% CI)	*p* value
Overall mortality						
Total	Female	3579	15,170	196	12.92 (11.17–14.86)	0.85
	Male	3579	15,225	193	12.68 (10.95–14.60)	
Age ≤50 years	Female	1502	6387	51	7.99 (5.95–10.50)	0.02
	Male	1827	8076	40	4.95 (3.54–6.75)	
Age >50 years	Female	2077	8783	145	16.51 (13.93–19.43)	0.02
	Male	1752	7149	153	21.40 (18.14–25.07)	
Non‐AF/cirrhosis	Female	3232	13,606	132	9.70 (8.12–11.51)	0.93
	Male	3234	13,663	134	9.81 (8.22–11.62)	
AF/cirrhosis	Female	347	1564	64	40.92 (31.52–52.26)	0.66
	Male	345	1562	59	37.77 (28.75–48.72)	
White	Female	1651	7466	114	15.27 (12.59–18.34)	0.15
	Male	1645	7664	96	12.53 (10.15–15.30)	
Hispanic	Female	712	2615	36	13.77 (9.64–19.06)	0.88
	Male	710	2560	34	13.28 (9.20–18.56)	
Asian	Female	851	3912	32	8.18 (5.59–11.55)	0.85
	Male	884	3846	33	11.55 (5.91–12.05)	
Black	Female	74	341	5	14.65 (4.76–34.19)	0.07
	Male	80	319	12	34.19 (19.45–65.76)	
Nonliver‐related mortality						
Total	Female	3579	15,170	176	11.60 (9.95–13.45)	0.89
	Male	3579	15,225	174	11.43 (9.79–13.26)	
Age ≤50 years	Female	1502	6387	46	7.20 (5.27–9.61)	0.05
	Male	1827	8076	38	4.71 (3.33–6.46)	
Age >50 years	Female	2077	8783	130	14.80 (12.37–17.58)	0.04
	Male	1752	7149	136	19.02 (15.96–22.50)	
Non‐AF/cirrhosis	Female	3232	13,606	127	9.33 (7.78–11.11)	0.97
	Male	3234	13,663	127	9.30 (7.75–11.06)	
AF/cirrhosis	Female	347	1564	49	31.33 (23.18–41.42)	0.84
	Male	345	1562	47	30.09 (22.11–40.01)	
White	Female	1651	7466	104	13.93 (11.38–16.88)	0.21
	Male	1645	7664	89	11.61 (9.33–14.29)	
Hispanic	Female	712	2615	29	11.09 (7.43–7.43)	0.83
	Male	710	2560	30	11.72 (7.91–16.73)	
Asian	Female	851	3912	32	8.18 (5.59–11.55)	0.75
	Male	884	3846	29	7.54 (5.05–5.05)	
Black	Female	74	341	4	11.72 (3.19–30.01)	0.08
	Male	80	319	10	31.37 (15.04–57.70)	
Liver‐related mortality						
Total	Female	3579	15,170	20	1.32 (0.81–2.04)	0.86
	Male	3579	15,225	19	1.25 (0.75–1.95)	
Age ≤50 years	Female	1502	6387	5	0.78 (0.25–1.83)	0.15
	Male	1827	8076	2	0.25 (0.03–0.89)	
Age >50 years	Female	2077	8783	15	1.71 (0.96–2.82)	0.35
	Male	1752	7149	17	2.38 (1.39–3.81)	
Non‐AF/cirrhosis	Female	3232	13,606	5	0.37 (0.12–0.86)	0.57
	Male	3234	13,663	7	0.51 (0.21–1.06)	
AF/cirrhosis	Female	347	1564	15	9.59 (5.37–15.82)	0.57
	Male	345	1562	12	7.68 (3.97–13.42)	
White	Female	1651	7466	10	1.34 (0.64–2.46)	0.43
	Male	1645	7664	7	0.91 (0.37–1.88)	
Hispanic	Female	712	2615	7	2.68 (1.08–1.08)	0.38
	Male	710	2560	4	1.56 (0.43–4.00)	
Asian	Female	851	3912	0	0.00	0.04
	Male	884	3846	4	1.04 (1.04–2.66)	
Black	Female	74	341	1	2.93 (2.93–16.32)	0.02
	Male	80	319	2	16.32 (0.76–22.67)	

*Note*: Data for other race and ethnicity was not included.

Abbreviations: AF, advanced fibrosis; MASLD, metabolic dysfunction‐associated steatotic liver disease; PSM, propensity score matching.

However, there were differences by age and sex in subgroup analyses (Table 2). In the group aged 50 years or younger, females had higher overall (7.99 vs. 4.95 per 1000 PY, *p *= 0.02) and nonliver‐related (7.20 vs. 4.71 per 1000 PY, *p *= 0.05) mortality rates compared with males. On the other hand, males had higher overall (21.40 vs. 16.51 per 1000 PY, *p *= 0.02) and nonliver‐related (19.02 vs. 14.80 per 1000 PY, *p *= 0.04) mortality rates compared with females in the group aged over 50 years. There was no significant difference in liver‐related mortality rate between males and females in both the group aged ≤50 years and the older group (0.25 vs. 0.78 per 1000 PY, *p *= 0.15 and 2.38 vs. 1.71 per 1000 PY, *p *= 0.35, respectively). In males and females aged 50 years or younger, the 5‐year cumulative incidence were 2.6 versus 3.4% for overall mortality, 2.3 versus 3.0% for nonliver‐related mortality, and 0.2 versus 0.44% for liver‐related mortality, respectively (Figure 2B). In the older group, the 5‐year cumulative incidence between males and females were 10.0 versus 7.3% for overall mortality, 9.4 versus 7.3% for nonliver‐related mortality, and 1.10 versus 0.57% for liver‐related mortality, respectively (Figure 2C). In the subgroup analyses of patients with or without advanced fibrosis/cirrhosis, the overall and cause‐specific mortality rates were all comparable between males and females (Figure S3). When stratified by race and ethnicity, there were no statistically significant differences between males and females in any of the racial ethnic subgroups though there was a trend for higher overall (34.19 vs. 14.65, *p *= 0.07) and nonliver‐related (31.37 vs. 11.72, *p *= 0.08) mortality rates in males as compared with females among Black patients (Table 2 and Figure S4). Number of events in liver‐related mortality analyses were very few for all subgroups.

### 2.3 Association Between Sex and Mortality

In the multivariable Cox regression model adjusted for age, hyperlipidemia, and CKD, there was no significant association between sex and the risk for overall (adjusted hazard ratio [aHR] 1.10, 95% confidence interval [CI] 0.90–1.35, *p *= 0.36), nonliver‐related (aHR 1.16, 95% CI 0.89–1.36, *p *= 0.39), or liver‐related (aHR 1.17, 95% CI 0.60–2.17, *p *= 0.68) mortality in the total PSM cohort (Table 3). However, significant interaction between sex and age was observed in Cox regression models for overall and nonliver‐related mortality (both *p* for interaction <0.05), but not for liver‐related mortality (Table S2). In further age‐stratified analyses, male (vs. female) sex was associated with about 40% lower risks of overall and nonliver‐related mortality (aHR 0.59, 95% CI 0.38–0.90, *p *= 0.01 and aHR 0.61, 95% CI 0.40–0.95, *p *= 0.03, respectively) in the younger group, whereas male (vs. female) sex was associated about 30% higher risks (aHR 1.32, 95% CI 1.05–1.66, *p *= 0.01 and aHR 1.30, 95% CI 1.02–1.66, *p *=  0.01, respectively) in the older group (Table 3). There was no significant association between sex and liver‐related mortality in both the age‐stratified subgroups (Table 3). The proportional hazard (PH) assumptions were valid for all multivariable Cox regression models including those of overall, nonliver‐related, and liver‐related mortality for the total PSM cohort as well as in age‐stratified subgroups with concordance index (C‐index) values ranging 0.65–0.82 (Table S3). Additionally, the majority of *E* values for aHRs ranged about 2–5, indicating that an unmeasured confounder would need to be associated with both sex and the outcome with an HR of at least 2–5 to fully explain away the observed association and thus suggesting that our results are robust including those for subgroup analyses (Table S4).

**TABLE 3:** Association between sex and mortality in patients with MASLD in the PSM cohort.

	Events, *n*	Univariable HR (95% CI)	*p* value	Multivariable HR^a^ (95% CI)	*p* value
Total					
Overall mortality					
Female	196	Reference		Reference	
Male	193	0.98 (0.80–1.20)	0.84	1.10 (0.90–1.35)	0.36
Nonliver‐related mortality					
Female	176	Reference		Reference	
Male	174	0.98 (0.80–1.21)	0.86	1.10 (0.89–1.36)	0.39
Liver‐related mortality					
Female	20	Reference		Reference	
Male	19	0.94 (0.50–1.76)	0.85	1.14 (0.60–2.17)	0.68
Age ≤50 years					
Overall mortality					
Female	51	Reference		Reference	
Male	40	0.62 (0.41–0.94)	0.02	0.59 (0.38–0.90)	0.01
Nonliver‐related mortality					
Female	46	Reference		Reference	
Male	38	0.65 (0.43–1.01)	0.05	0.61 (0.40–0.95)	0.03
Liver‐related mortality					
Female	5	Reference		Reference	
Male	2	0.31 (0.06–1.61)	0.16	0.32 (0.06–1.69)	0.18
Age >50 years					
Overall mortality					
Female	145	Reference		Reference	
Male	153	1.29 (1.03–1.62)	0.03	1.32 (1.05–1.66)	0.02
Nonliver‐related mortality					
Female	130	Reference		Reference	
Male	136	1.27 (1.00–1.62)	0.05	1.30 (1.02–1.66)	0.03
Liver‐related mortality					
Female	15	Reference		Reference	
Male	17	1.44 (0.72–2.89)	0.30	1.51 (0.74–3.05)	0.26

Abbreviations: HR, hazard ratio; MASLD, metabolic dysfunction‐associated steatotic liver disease; PSM, propensity score matching.

^a^ Adjusted for age, hyperlipidemia, and chronic kidney disease.

When using Fine‐Gray models to account for competing risks of liver‐related and nonliver‐related mortality, the results remained consistent with those of the Cox regression analyses (Table S5). Consistent findings were also observed in additional sensitivity analyses after excluding patients with sex‐specific cancers (breast, cervix, ovarian, and endometrial cancers for females; prostate and testicular cancers for males) at baseline (Table 4). In the total cohort, there was no significant association between sex and risks for overall (aHR 1.20, 95% CI 0.97–1.48, *p *= 0.09), nonliver‐related (aHR 1.20, 95% CI 0.97–1.52, *p *= 0.09), or liver‐related (aHR 1.10, 95% CI 0.57–2.13, *p *= 0.77) mortality; in the patients aged >50 years, male sex remained significantly associated with about 40% higher risks of overall (aHR 1.41, 95% CI 1.11–1.80, *p *= 0.01) and nonliver‐related (aHR 1.41, 95% CI 1.09–1.82, *p *= 0.01) mortality; and in the patients aged ≤50 years, male (vs. female) sex showed a trend toward lower risks of overall (aHR 0.67, 95% CI 0.43–1.04, *p *= 0.07) and nonliver‐related (aHR 0.72, 95% CI 0.45–1.14, *p *= 0.16) mortality.

**TABLE 4:** Sensitivity analyses for Cox regression models, after excluding patients with sex‐specific cancers from the PSM cohort.

	Events, *n*	Univariable HR (95% CI)	*p* value	Multivariable HR^a^ (95% CI)	*p* value
Total					
Overall mortality					
Female	170	Reference		Reference	
Male	189	1.07 (0.87–1.31)	0.54	1.20 (0.97–1.48)	0.09
Nonliver‐related mortality					
Female	151	Reference		Reference	
Male	171	1.08 (0.87–1.35)	0.46	1.21 (0.97–1.52)	0.09
Liver‐related mortality					
Female	19	Reference		Reference	
Male	18	0.91 (0.48–1.73)	0.76	1.10 (0.57–2.13)	0.77
Age ≤50 years					
Overall mortality					
Female	42	Reference		Reference	
Male	40	0.73 (0.47–1.12)	0.15	0.67 (0.43–1.04)	0.07
Nonliver‐related mortality					
Female	37	Reference		Reference	
Male	38	0.78 (0.50–1.23)	0.29	0.72 (0.45–1.14)	0.16
Liver‐related mortality					
Female	5	Reference		Reference	
Male	2	0.31 (0.06–1.57)	0.16	0.31 (0.06–1.62)	0.16
Age >50 years					
Overall mortality					
Female	128	Reference		Reference	
Male	149	1.38 (1.09–1.75)	0.01	1.41 (1.11–1.80)	0.01
Nonliver‐related mortality					
Female	114	Reference		Reference	
Male	113	1.38 (1.08–1.77)	0.01	1.41 (1.09–1.82)	0.01
Liver‐related mortality					
Female	14	Reference		Reference	
Male	16	1.39 (0.68–2.85)	0.37	1.48 (0.71–3.06)	0.30

Abbreviations: HR, hazard ratio; MASLD, metabolic dysfunction‐associated steatotic liver disease; PSM, propensity score matching.

^a^ Adjusted for age, hyperlipidemia, and chronic kidney disease.

## 3 Discussion

In this real‐world longitudinal cohort study of MASLD patients with PSM to balance background characteristics of the two sex subgroups, we found that the overall, nonliver‐related, and liver‐related mortality rates were similar between males and females overall. However, in subgroup analyses by age, sex differences in the risk of mortality were evident with females aged 50 years or younger having about 40% higher risk of overall and nonliver‐related mortality while males older than 50 years having about 30% higher risks. These findings underscore the importance of sex and age consideration in the management strategies of patients with MASLD.

Our findings of higher overall and nonliver‐related mortality in females as compared with males in the cohort aged 50 years or younger may be somewhat unexpected as one may have expected that the protective effect of estrogen in premenopausal woman should have helped decrease the risk of nonliver comorbidities such as CVD [11, 12]. With the protection of estrogen, females are usually at less risk of CVD than males until menopause when the protective effects from estrogen are lost and females and males become more equal in their risk of mortality especially related to CVD [13, 14]. In the United States, the average age for menopause is 52 years old, but some females may experience premature menopause (<40 years old) or early menopause (40–49 years old), which can place them at higher risk for various comorbidities including CVD, cerebrovascular accidents, dementia, and osteoporosis at an earlier age [11, 12]. Prior studies have reported significant association between earlier onset of menopause with tobacco or alcohol consumption, unhealthy diet, overweight or obesity (vs. normal weight), presence of diabetes, especially for women with diabetes who were at 3.5 times higher risk of experiencing premature menopause [15, 16, 17], and it is known that approximately 6% of females every year enter into either premature (1%) or early (5%) menopause [18]. Therefore, we conjectured that some of the females may have entered into early or premature menopause placing them at higher risk for nonliver comorbidities including CVD and CVD is the number one cause of death of MASLD patients [19], which may provide one suggestion as to why younger (≤50 years) females had higher mortality rate and risk than males. Such a suggestion is reasonable as studies have shown that young females with acute coronary syndromes or myocardial infarction have significant higher mortality compared with young males, even after adjustment for medications and other coexisting comorbidities [20, 21]. Moreover, prior studies have reported more pronounced association between MASLD (vs. non‐MASLD) and increased risk of sudden cardiac arrest and heart failure in young females, with 2.27‐fold and 4.03‐fold increased risk for sudden cardiac arrest and heart failure in young women, whereas 1.47‐fold and 1.69‐fold in young men [22, 23]. Combined these results highlight the importance of optimizing the prevention, monitoring, and therapeutic strategies for nonliver adverse events in young females with MASLD, especially those who may experiencing premature or early menopause.

Our findings that males experienced a 32% higher risk of overall mortality, specifically a 30% higher risk of nonliver‐related mortality, in MASLD patients aged over 50 years are similar to findings from prior reports. One such study reported that males aged over 60 years with MASLD had a 56% higher risk of all‐cause mortality compared with females of the same age in their population‐based US cohort [24], while another US nationwide study reported higher risk of CVD and CKD development in male patients with MASLD [25]. Other studies using global data and multicenter clinical cohort have reported similar results as well [26, 27].

There were several strengths in this study. First, our study patients with MASLD were consecutively included from all clinics at the university medical center, most of whom were not from specialized liver clinics and were identified primarily through imaging and liver disease severity was estimated using FIB‐4 index making the study population more representative of the real‐world MASLD patients. Second, we used PSM method to balance baseline characteristics between the two study groups to minimize the effects of confounders and comorbidities. Third, we applied the updated MASLD diagnostic criteria as the inclusion criteria, which will help the generalizability of our findings for future studies of steatotic liver disease (SLD). We also acknowledge several limitations. Mortality data confirmed by the National Death Index (NDI) might involve misclassification of the cause of death, but one of our major findings and outcomes were overall mortality, which would not be affected by this. The liver‐related mortality data should be interpreted with caution due to the small number of liver‐related deaths, especially in subgroup analyses. However, the small number of liver‐related deaths in our study is in line with prior knowledge that the number one cause of death among people with MASLD is nonliver‐related and specifically CVD‐related [19, 28]. Our study lacked detailed data on treatment regimens for CVD risk factors such as hypertension, hyperlipidemia, and diabetes, including lifestyle interventions such as diet and exercise, all of which could potentially influence the mortality risk of patients with MASLD [29]. Therefore, further studies with evaluations of these variables are needed, and a causal conclusion cannot be drawn from the current study association analyses given the potential presence of unmeasured confounding factors. Future studies with larger sample size are also needed to validate our findings, especially those of subgroup analyses without Bonferroni correction.

In conclusion, this study provides evidence of significant sex differences in the risk of mortality among age‐stratified patients with MASLD using real‐world longitudinal cohort data. In the younger (≤50 years) group, females had about 40% higher risk of overall mortality compared with males, while in the group older than 50 years, males had about 30% higher risk of both overall and nonliver‐related mortality compared with females. Our study provides strong data to support sex‐ and age‐based strategies in the prevention, monitoring, and treatment of people with MASLD.

## 4 Materials and Methods

### 4.1 Study Design and Population

This is a retrospective cohort study of adults (≥18 years) with MASLD who were seen between 1995 and 2023 at any general or subspecialty clinics of the Stanford Healthcare system, which includes hospitals and clinics at 22 locations throughout the San Francisco Bay Area, California, USA. We identified consecutive patients with SLD via imaging or liver histology (Figure **1**). All patients had at least one of five cardiometabolic risk factors: (1) body mass index (BMI) ≥25 kg/m^2^ (23 kg/m^2^ for Asian) or waist circumference >94 cm (male), >80 cm (female); (2) fasting serum glucose ≥5.6 mmol/L (100 mg/dL), 2‐h postload glucose levels ≥7.8 mmol/L (140 mg/dL), HbA1c ≥5.7% (39 mmol/L), type 2 diabetes or treatment for type 2 diabetes; (3) blood pressure ≥130/85 mmHg or specific antihypertension drug treatment; (4) plasma triglycerides ≥1.7 mmol/L (150 mg/dL) or lipid lowering treatment; or (5) plasma high‐density lipoprotein cholesterol ≤1.0 mmol/L (40 mg/dL) (male) and ≤1.3 mmol/L (50 mg/dL) (female) or lipid lowering treatment [30]. We excluded the patients if they (1) were younger than 18 years, (2) had significant alcohol use; (3) had a diagnosis of HCC before MASLD diagnosis; (4) lacked FIB‐4 index, which is calculated from age, ALT, AST and platelet (PLT); or (5) had no follow‐up after MASLD diagnostic date (Figure 1). All patients who met the diagnostic criteria of MASLD were included, regardless of presence of pharmacologic interventions predisposing patients to metabolic disorders. Baseline demographics, BMI, comorbidities including hypertension, hyperlipidemia, diabetes, CVD, CKD and nonliver cancers, CCI, presence of viral hepatitis and cirrhosis, and laboratory test data were collected via individual patient chart review. The study was approved by the Institutional Review Board at Stanford University, Stanford, California, USA (ethics approval number: 13927).

### 4.2 Study Outcomes and Definitions

Primary study outcomes were (1) overall, (2) nonliver‐related, and (3) liver‐related mortality. Death data were confirmed via chart review and supplemented by NDI search for patients who may have died in other healthcare system in other locations of the United States. Mortality was considered to be liver‐related if the primary cause of death was related to a complication of hepatic failure and/or of end‐stage liver disease. Nonliver‐related mortality was defined as deaths attributed to nonliver cancers or other causes not related to end‐stage liver disease. We used the FIB‐4 index to determine liver disease severity as recommended by practice guidelines [31, 32]. FIB‐4 index was calculated using the formula: FIB‐4 = age (years) × AST (U/L)/PLT (10^9^/L) × ALT^1/2^ (U/L)]. Baseline FIB‐4 index was calculated using ALT, AST, and PLT data tested within 6 months of the MASLD diagnosis date. Patients with FIB‐4 index >2.67 were considered to have advanced fibrosis (fibrosis Stage 3–4) in MASLD [33]. Cirrhosis was determined by clinical evidence of esophageal varices, ascites, or hepatic encephalopathy, imaging findings of nodular liver contour, and splenomegaly, noninvasive methods such as Fibroscan, magnetic resonance elastography, shear wave ultrasound, or Fibrosure, or liver histology. The date of first medical encounter with MASLD diagnosis was considered the index date for mortality analysis, with censoring criteria including death, loss to follow‐up, or completion of the 15‐year follow‐up period, whichever occurred first.

### 4.3 Statistical Analysis

Continuous variables were reported as mean and standard deviation (SD) if normally distributed or median and interquartile ranges if not. Categorical data were reported as the numbers and percentages. Patients were classified into two study groups: males and females. Differences between groups were compared using the *t* test or Wilcoxon rank‐sum test for continuous variables and chi‐squared test for categorical variables. To reduce the effects of confounding variables, we used PSM to balance the baseline characteristics between the female and male groups. Propensity scores were estimated using a logistic regression model that included race and ethnicity, BMI, diabetes, CCI, FIB‐4 index, and the presence of viral hepatitis. A 1:1 nearest‐neighbor matching without replacement was performed, using a caliper of 0.1 of the SD of the logit propensity scores. Although a caliper width of 0.2 is commonly recommended as a general rule for PSM, a more stringent caliper of 0.1 was applied in this study to further improve covariate balance and minimize residual confounding [34, 35, 36]. We used the standardized mean difference (SMD) to evaluate the balancing of potential confounding variables between the male and female groups, as *p* values are sensitive to sample size. Variables with SMD <0.1 were considered to be balanced between groups. Additionally, visual comparisons of the propensity score distributions before and after matching was performed to assess matching quality.

Mortality rates per 1000 PY were estimated by sex and compared between the two groups. Kaplan–Meier method was used to examine the cumulative incidence of mortality, with log‐rank test for the comparison between two groups. We also estimated mortality rates by sex in subgroups stratified by age, race and ethnicity, and liver disease severity, as these factors have been reported to influence mortality in patients with MASLD [37, 38, 39]. Univariable and multivariable Cox PHs regression analyses were performed to estimate unadjusted and aHR for risk of mortality. Covariates with SMD of 0.1 or higher after PSM with potential significant clinical impacts were included as covariates in multivariable Cox regression models. We performed PHs assumption testing and calculated C‐index values for multivariable Cox regression models to assess model validity and predictive performance. We also calculated *E* values to quantify the minimum strength of association that an unmeasured confounder would need to have with both the exposure and the outcome to fully explain away the observed association, conditional on the measured covariates [40]. We used the Fine‐Gray competing risk models to derive cause‐specific subdistribution hazard ratios for risks of nonliver‐related and liver‐related mortality. Given the potential influence of sex‐specific cancers on the findings of sex disparities in mortality, we performed sensitivity analyses excluding patients with sex‐specific cancers at baseline. Statistically significant was defined as two‐tailed *p *< 0.05. All analysis were conducted using R version 4.1.1 (R Foundation for Statistical Computing, Vienna, Austria).

## Author Contributions

Mindie H. Nguyen developed the idea. Taotao Yan and Mindie H. Nguyen designed the study. Taotao Yan, Nicholas Chien, Vy H. Nguyen, Isaac Le, Surya Teja Gudapati, Angela Chau, and Xinrong Zhang collected the data. Taotao Yan, Nicholas Chien, and Mindie H. Nguyen have full access to all the data in the study and take responsibility for the integrity of the data and the accuracy of the data analysis. Taotao Yan and Mindie H. Nguyen drafted the manuscript. Taotao Yan, Scott Barnett, Sovann Linden, Linda Henry, Ramsey Cheung, and Mindie H. Nguyen made critical revisions to the manuscript. Mindie H. Nguyen supervised the study. All authors have read and approved the final manuscript.

## Funding

The authors have nothing to report.

## Conflicts of Interest

Mindie H. Nguyen received research grants via Stanford University from Pfizer, Enanta, Astra Zeneca, GSK, Delfi, Innogen, Exact Science, CurveBio, Gilead, Vir Biotech, Helio Health, National Institute of Health, and personal fees from consulting/advisory board from Exelixis, Gilead, and GSK. All the other authors declare no conflicts of interest.

## Ethics Statement

The study was approved by the Institutional Review Board at Stanford University, Stanford, California, USA (approval no. 13927).

## Supporting information




**Table S1**: Incidence of mortality in patients with MASLD in the total unmatched cohort.
**Table S2**: Interaction analyses between age and sex on mortality pf patients with MASLD in the total PSM cohort.
**Table S3**: Proportional hazard assumption tests and concordance index of the multivariable Cox regression models.
**Table S4**: *E* values for adjusted hazard ratios relating to sex with mortality in multivariable Cox regression models.
**Table S5**: Fine‐Gray competing risk models for cause‐specific mortality in patients with MASLD in the PSM cohort.
**Figure S1**: Cumulative incidence of mortality in patients with MASLD in the total unmatched cohort by sex.
**Figure S2**: Density distribution of propensity scores between female and male groups (A) before and (B) after PSM.
**Figure S3**: Cumulative incidence of mortality in patients with MASLD by sex in the subgroup of patients (A) without advanced fibrosis/cirrhosis and (B) with advanced fibrosis/cirrhosis within the PSM cohort.
**Figure S4**: Cumulative incidence of mortality in patients with MASLD by sex in the subgroup of (A) White (B) Hispanic (C) Asian and (D) Black patients within the PSM cohort.

## Data Availability

All data supporting this study's results are included in the article and the Supporting Information.
