# Supplementary material for: Differential Mortality Risk by Age in Males and Females With Steatotic Liver Disease
Source: MedComm (2020). 2026 Apr 5;7(4):e70677. doi: 10.1002/mco2.70677 (PMC13051813; doi:10.1002/mco2.70677)
Supplement: Supplementary file 1 — Table S1: Incidence of mortality in patients with MASLD in the total unmatched cohort. Table S2: Interaction analyses between age and sex on mortality pf patients with MASLD in the total PSM cohort. Table S3: Proportional hazard assumption tests and concordance index of the multivariable Cox regression models. Table S4: E values for adjusted hazard ratios relating to sex with mortality in multivariable Cox regression models. Table S5: Fine‐Gray competing risk models for cause‐specific mortality in patients with MASLD in the PSM cohort. Figure S1: Cumulative incidence of mortality in patients with MASLD in the total unmatched cohort by sex. Figure S2: Density distribution of propensity scores between female and male groups (A) before and (B) after PSM. Figure S3: Cumulative incidence of mortality in patients with MASLD by sex in the subgroup of patients (A) without advanced fibrosis/cirrhosis and (B) with advanced fibrosis/cirrhosis within the PSM cohort. Figure S4: Cumulative incidence of mortality in patients with MASLD by sex in the subgroup of (A) White (B) Hispanic (C) Asian and (D) Black patients within the PSM cohort. [file MCO2-7-e70677-s001.docx]

**Supplementary Material**

**Title:** Differential Mortality Risk by Age in Males and Females with Steatotic Liver Disease

Taotao Yan^1,2^, Nicholas Chien^1^, Vy H. Nguyen^1^, Isaac Le^1^, Surya Teja Gudapati^1^, Angela Chau^1^, Xinrong Zhang^1^, Scott Barnett^1^, Sovann Linden^1^, Linda Henry^1^, Ramsey Cheung^1,3^, Mindie H. Nguyen^*1,4,5^

1) Division of Gastroenterology and Hepatology, Stanford University Medical Center, Palo Alto, California, USA

2) Department of Infectious Diseases, The First Affiliated Hospital of Xi’an Jiaotong University, Xi’an, Shaanxi, China

3) Division of Gastroenterology and Hepatology, Veterans Affairs Palo Alto Healthcare System, Palo Alto, California, USA

4) Department of Epidemiology and Population Health, Stanford University Medical Center, Palo Alto, California, USA

5) Stanford Cancer Institute, Stanford University Medical Center, Palo Alto, California, USA

**Correspondence and reprint request:**

Mindie H Nguyen, MD, MAS

Professor of Medicine (Gastroenterology and Hepatology) and, by courtesy, of Epidemiology and Population Health

Stanford University Medical Center, Palo Alto, California

Email: mindiehn@stanford.edu

**Table of contents**

| Table of contents | Page 2 |
| --- | --- |
| Table S1. Incidence of mortality in patients with MASLD in the total unmatched cohort. | Page 3 |
| Table S2. Interaction analyses between age and sex on mortality pf patients with MASLD in the total PSM cohort. | Page 4 |
| Table S3. Proportional hazard assumption tests and concordance index of the multivariable Cox regression models. | Page 5 |
| Table S4. E-values for adjusted hazard ratios relating to sex with mortality in multivariable Cox regression models. | Page 6 |
| Table S5. Fine-Gray competing risk models for cause-specific mortality in patients with MASLD in the PSM cohort. | Page 7 |
| Figure S1. Cumulative incidence of mortality in patients with MASLD in the total unmatched cohort by sex. | Page 8 |
| Figure S2. Density distribution of propensity scores between female and male groups (A) before and (B) after PSM. | Page 9 |
| Figure S3. Cumulative incidence of mortality in patients with MASLD by sex in the subgroup of patients (A) without advanced fibrosis/cirrhosis and (B) with advanced fibrosis/cirrhosis within the PSM cohort. | Page 10 |
| Figure S4. Cumulative incidence of mortality in patients with MASLD by sex in the subgroup of (A) White (B) Hispanic (C) Asian and (D) Black patients within the PSM cohort. | Page 11 |

**Table S1. Incidence of mortality in patients with MASLD in the total unmatched cohort.**

| **Outcome** | **Patient**  **No.** | **Person-**  **years** | **Event,**  **n** | **Mortality rate per 1000**  **person-years, (95% CI)** | ***P***  **value** |
| --- | --- | --- | --- | --- | --- |
| **Overall mortality** | | | | | |
| Female | 4521 | 19016 | 256 | 13.46 (11.86-15.22) | 0.21 |
| Male | 3996 | 17209 | 206 | 11.97 (10.39-13.72) |  |
| **Nonliver-related mortality** | | | | | |
| Female | 4521 | 19016 | 233 | 12.25 (10.73-13.93) | 0.15 |
| Male | 3996 | 17209 | 183 | 10.63 (9.15-12.29) |  |
| **Liver-related mortality** | | | | | |
| Female | 4521 | 19016 | 23 | 1.21 (0.77-1.82) | 0.73 |
| Male | 3996 | 17209 | 23 | 1.34 (0.85-2.01) |  |

Abbreviation: MASLD, Metabolic dysfunction-associated steatotic liver disease; CI, Confidence interval.

**Table S2. Interaction analyses between age and sex on mortality pf patients with MASLD in the total PSM cohort.**

| **Outcome** | **Univariable HR (95% CI)** | ***P* value** | ***P* for interaction** | **Multivariable HR^a^ (95% CI)** | ***P* value** | ***P* for interaction** |
| --- | --- | --- | --- | --- | --- | --- |
| **Overall mortality** | | | | | | |
| Female and age ≤50 years | Reference |  | 0.002 | Reference |  | 0.01 |
| Female and age >50 years | 2.07 (1.50-2.84) | 0.02 |  | 1.99 (1.44-2.75) | <0.001 |  |
| Male and age ≤50 years | 0.62 (0.41-0.94) | <0.001 |  | 0.61 (0.40-0.93) | 0.02 |  |
| Male and age >50 years | 2.67 (1.95-3.67) | <0.001 |  | 2.28 (1.63-3.17) | <0.001 |  |
| **Nonliver-related mortality** | | | | | | |
| Female and age ≤50 years | Reference |  | 0.01 | Reference |  | 0.02 |
| Female and age >50 years | 2.05 (1.46-2.87) | <0.001 |  | 1.92 (1.37-2.71) | <0.001 |  |
| Male and age ≤50 years | 0.65 (0.43-1.01) | 0.05 |  | 0.64 (0.41-0.98) | 0.04 |  |
| Male and age >50 years | 2.62 (1.88-3.67) | <0.001 |  | 2.17 (1.53-3.08) | <0.001 |  |
| **Liver-related mortality** | | | | | | |
| Female and age ≤50 years | Reference |  | 0.10 | Reference |  | 0.14 |
| Female and age >50 years | 2.21 (0.80-6.09) | 0.12 |  | 2.55 (0.91-7.10) | 0.07 |  |
| Male and age ≤50 years | 0.31 (0.06-1.61) | 0.16 |  | 0.34 (0.06-1.73) | 0.19 |  |
| Male and age >50 years | 3.11 (1.15-8.46) | 0.03 |  | 3.29 (1.17-9.25) | 0.02 |  |

^a^Adjusted for hyperlipidemia and chronic kidney disease.

Abbreviation: MASLD, Metabolic dysfunction-associated steatotic liver disease; PSM, Propensity score matching; HR, Hazard ratio; CI, Confidence interval.

**Table S3. Proportional hazard assumption tests and concordance index of the multivariable Cox regression models.**

| **Outcome** | **χ²** | ***P* value** | **C-index** |
| --- | --- | --- | --- |
| **Total** | | | |
| Overall mortality | 1.89 | 0.17 | 0.73 |
| Nonliver-related mortality | 0.48 | 0.49 | 0.72 |
| Liver-related mortality | 2.85 | 0.09 | 0.82 |
| **Age ≤50 years** | | | |
| Overall mortality | 0.02 | 0.89 | 0.66 |
| Nonliver-related mortality | 0.001 | 0.98 | 0.65 |
| Liver-related mortality | 0.001 | 0.98 | 0.81 |
| **Age >50 years** | | | |
| Overall mortality | 1.93 | 0.16 | 0.72 |
| Nonliver-related mortality | 0.46 | 0.50 | 0.71 |
| Liver-related mortality | 2.66 | 0.10 | 0.82 |

All Cox regression models include age, hyperlipidemia, and chronic kidney disease as covariates.

**Table S4. E-values for adjusted hazard ratios relating to sex with mortality in multivariable Cox regression models.**

| **Outcome** | Multivariable  adjusted HR^a^(95% CI) | E-value for  adjusted HR | E-value for limit of CI closest to 1 |
| --- | --- | --- | --- |
| **Total** | | | |
| Overall mortality | 1.10 (0.90-1.35) | 1.43 | 1 |
| Nonliver-related mortality | 1.10 (0.89-1.36) | 1.43 | 1 |
| Liver-related mortality | 1.14 (0.60-2.17) | 2.15 | 1 |
| **Age ≤50 years** | | | |
| Overall mortality | 0.59 (0.38-0.90) | 2.78 | 1.46 |
| Nonliver-related mortality | 0.61 (0.40-0.95) | 2.66 | 1.29 |
| Liver-related mortality | 0.32 (0.06-1.69) | 5.70 | 1 |
| **Age >50 years** | | | |
| Overall mortality | 1.32 (1.05-1.66) | 1.97 | 1.28 |
| Nonliver-related mortality | 1.30 (1.02-1.66) | 1.92 | 1.16 |
| Liver-related mortality | 1.51 (0.74-3.05) | 2.39 | 1 |

^a^Adjusted for age, hyperlipidemia, and chronic kidney disease.

Abbreviations: HR, Hazard ratio, CI, confidence interval.

**Table S5. Fine-Gray competing risk models for cause-specific mortality in patients with MASLD in the PSM cohort.**

| **Outcome** | **Events, n** | **Univariable**  **sHR (95% CI)** | ***P* value** | **Multivariable**  **sHR^a^ (95% CI)** | ***P* value** |
| --- | --- | --- | --- | --- | --- |
| **Total** | | | | | |
| ***Nonliver-related mortality*** | | | | | |
| Female | 176 | Reference |  | Reference |  |
| Male | 174 | 0.98 (0.80-1.21) | 0.88 | 1.09 (0.88-1.35) | 0.42 |
| ***Liver-related mortality*** | | | | | |
| Female | 20 | Reference |  | Reference |  |
| Male | 19 | 0.94 (0.51-1.77) | 0.86 | 1.12 (0.56-2.21) | 0.75 |
| **Age ≤50 years** | | | | | |
| ***Nonliver-related mortality*** | | | | | |
| Female | 46 | Reference |  | Reference |  |
| Male | 38 | 0.66 (0.43-1.01) | 0.05 | 0.62 (0.40-0.96) | 0.03 |
| ***Liver-related mortality*** | | | | | |
| Female | 5 | Reference |  | Reference |  |
| Male | 2 | 0.32 (0.06-1.66) | 0.17 | 0.33 (0.06-1.91) | 0.21 |
| **Age >50 years** | | | | | |
| ***Nonliver-related mortality*** | | | | | |
| Female | 130 | Reference |  | Reference |  |
| Male | 136 | 1.27 (1.00-1.62) | 0.05 | 1.29 (1.01-1.64) | 0.04 |
| ***Liver-related mortality*** | | | | | |
| Female | 15 | Reference |  | Reference |  |
| Male | 17 | 1.38 (0.70-2.76) | 0.35 | 1.44 (0.68-3.03) | 0.34 |

^a^Adjusted for hyperlipidemia and chronic kidney disease.

Abbreviation: MASLD, Metabolic dysfunction-associated steatotic liver disease; PSM, Propensity score matching; sHR, Subdistribution hazard ratio; CI, Confidence interval.

**Figure S1. Cumulative incidence of mortality in patients with MASLD in the total unmatched cohort by sex.**

**
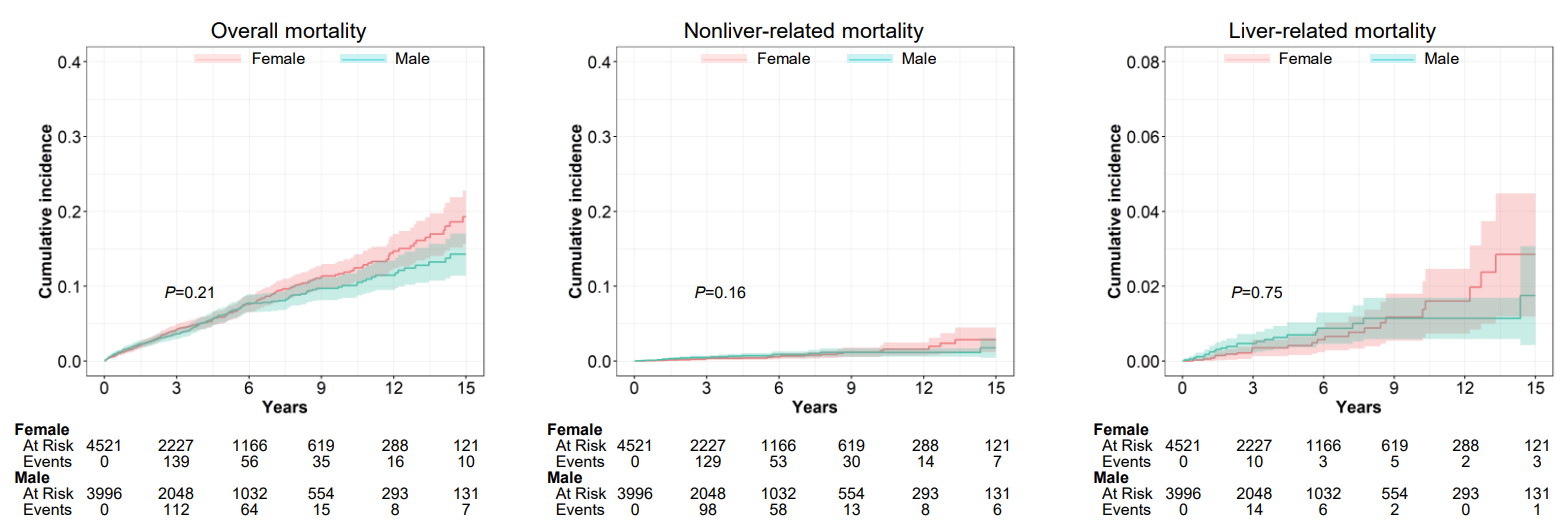
**

Abbreviation: MASLD, Metabolic dysfunction-associated steatotic liver disease.

**Figure S2. Density distribution of propensity scores between female and male groups (A) before and (B) after PSM.**


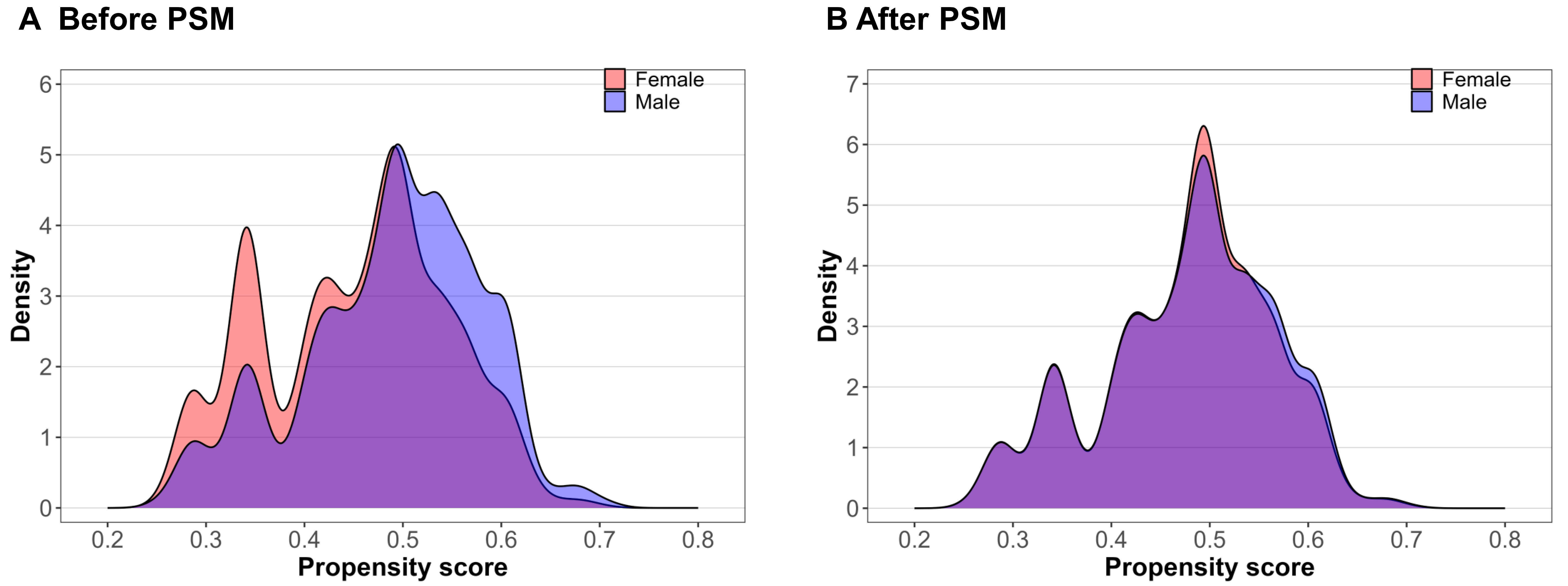


Abbreviation: PSM, Propensity score matching.

**Figure S3. Cumulative incidence of mortality in patients with MASLD by sex in the subgroup of patients (A) without advanced fibrosis/cirrhosis and (B) with advanced fibrosis/cirrhosis within the PSM cohort.**


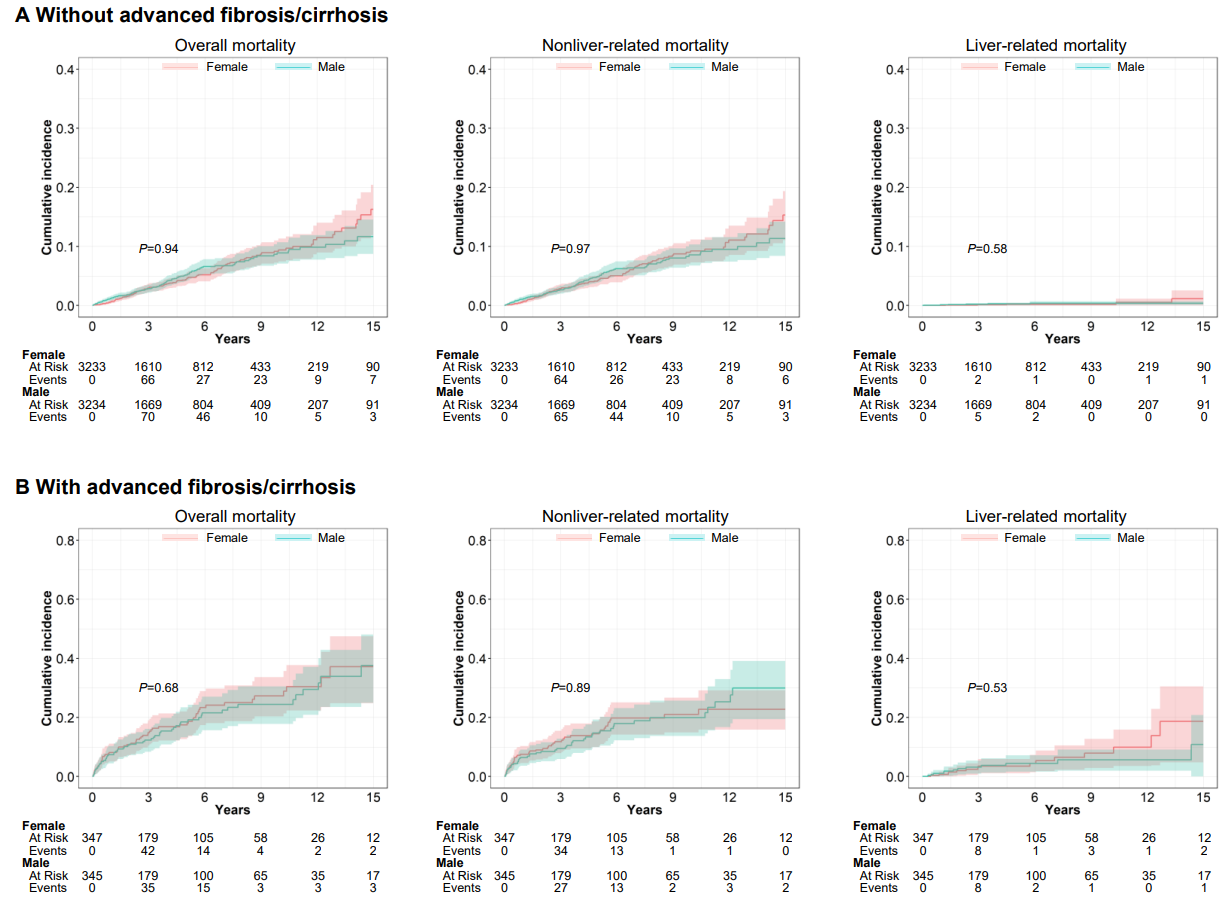


Abbreviation: MASLD, Metabolic dysfunction-associated steatotic liver disease; PSM, Propensity score matching.

**Figure S4. Cumulative incidence of mortality in patients with MASLD by sex in the subgroup of (A) White (B) Hispanic (C) Asian and (D) Black patients within the PSM cohort.**

**
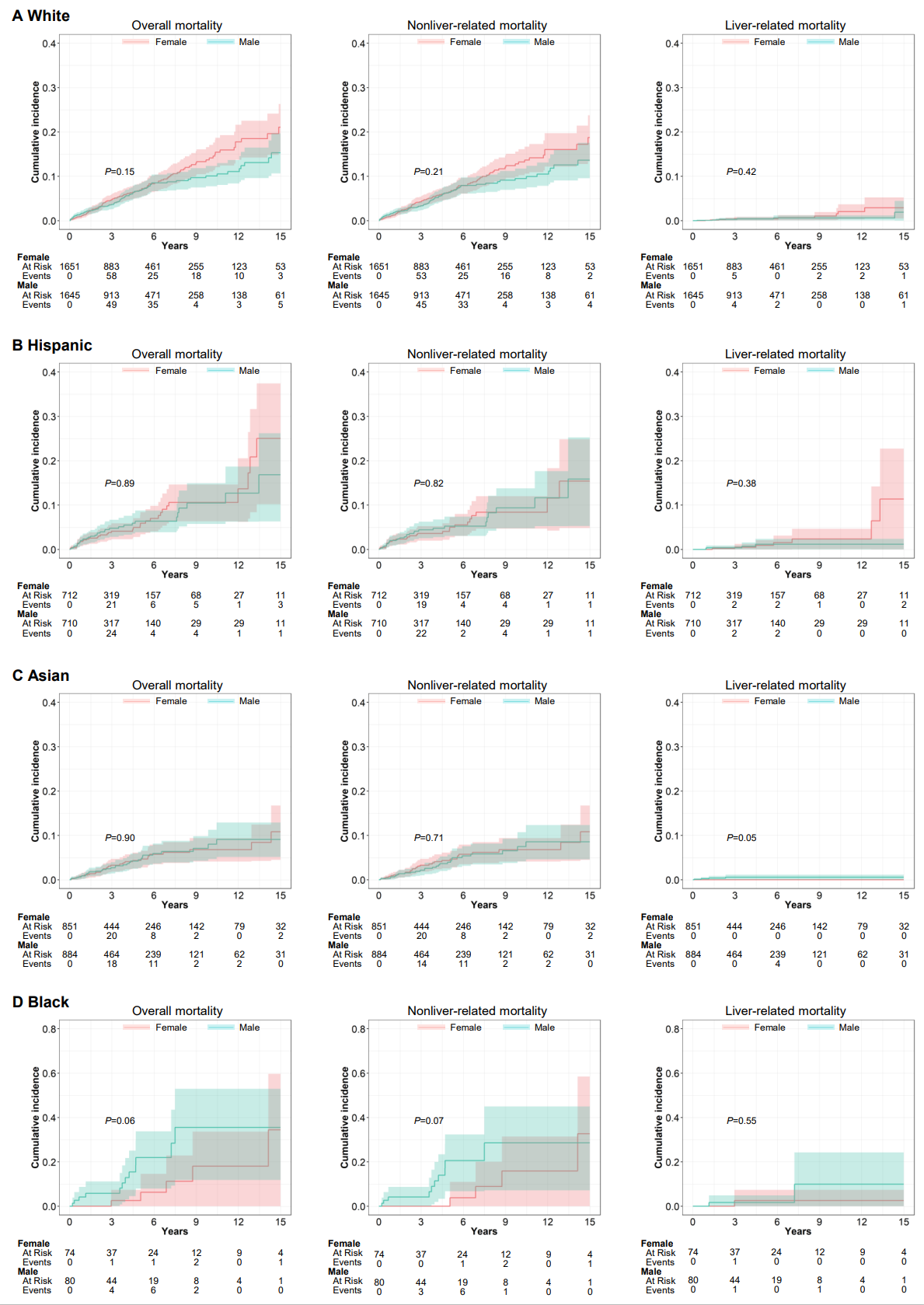
**

Abbreviation: MASLD, Metabolic dysfunction-associated steatotic liver disease; PSM, Propensity score matching.
